# Validity and reliability of the Urdu version of the 5D itching scale to assess pruritus among patients with chronic kidney disease in Pakistan

**DOI:** 10.1186/s12882-017-0717-0

**Published:** 2017-10-02

**Authors:** Inayat Ur Rehman, Tahir Mehmood Khan

**Affiliations:** School of Pharmacy, Monash University, Jalan Lagoon Selatan, Bandar Sunway, Selangor Malaysia

**Keywords:** Chronic kidney disease, End stage renal disease, Pruritus, Pakistan, 5D itch scale

## Abstract

**Background:**

Chronic kidney disease (CKD) has become a major public health issue worldwide in the past few years. Pruritus is a common, well-recognized complication often seen in patients with chronic renal failure. For assessment of pruritus, different tools are used but these tools are unable to identify the changes and variations in the severity of pruritus. The aim of our study was to validate the Urdu-version of the Urdu-version of the 5D itch scale among patients suffering from chronic kidney disease in Pakistan.

**Method:**

The 5D itch scale was translated from English into Urdu following translation guidelines for translation. Face and content validity was determined by a panel of experts and piloted. For retest, the Urdu version of the 5D itch scale was administered at baseline and two weeks.

**Results:**

A total of 50 participants with end stage renal disease were recruited, and of these, 64% were males. Exploratory factor analysis revealed that the 5D–IS had 2-factor loadings: “Pattern and activity” and “Distribution” with Kaiser–Mayer–Olkin (KMO) = 0.802, Bartlett’s test of sphericity was significant (*df* = 28, *p* < 0.001). At test re-test, Cronbach’s alpha was 0.914, while the intra class correlation was 0.9160 (95% confidence interval 0.941–0.975), which is a highly significant correlation (*p* < 0.0001).

**Conclusion:**

The Urdu version of the 5D itch scale was found to be a valid and reliable instrument for assessing pruritus and its severity in patients with chronic kidney disease in Pakistan.

**Electronic supplementary material:**

The online version of this article (10.1186/s12882-017-0717-0) contains supplementary material, which is available to authorized users.

## Background

Chronic kidney disease (CKD) has become a major public health issue worldwide in the past few years, owing to its increased prevalence and incidence [1, 2]. In 2013, CKD was directly linked as the cause of approximately 956,200 deaths worldwide, 134.6% more than that recorded in 1990 [3]. It is due to this reason that CKD has increased in ranking in terms of highest mortality rate, from 27th in 2010 [4] to 19th in 2013 [5]. The incidence of chronic kidney disease is reported to be higher in South Asian populations as compared to European populations [6, 7]. In Pakistan, CKD is on the rise, with multiple factors being cited, for instance poor availability of health care, a faulty primary health care system, inadequate health education, insufficient government financial support, and high rates of diabetes and hypertension [8]. In addition, the lack of a national-level department in Pakistan for registering kidney diseases makes it next to impossible to assess CKD cases, the no of patients on dialysis, mortality rates, and subsequently, how funds should be allocate [8]. A recent study published in 2014 regarding the prevalence of chronic kidney disease in Pakistan suggested a prevalence rate of 12.5% [9].

Pruritus adds to the complications of patients with CKD. It is well recognized as a common complication in patients with chronic renal failure [10–12]. Pruritus is an unpleasant sensation, accompanied by a desire to scratch oneself at the affected areas in order to obtain relief [13]. Pruritus is not only graded as the most common, but also incapacitating complication that has major long-standing effects on the patient’s quality of life, and prevalent in patients with end stage renal disease (ESRD) undergoing peritoneal dialysis (PD) and Hemodialysis (HD) [14–18]. At any point in time almost every patient undergoing dialysis suffers from pruritus, irrespective of the severity of it [19], and has been reported in 22% to 84% of patients in ESRD who have undergone hemodialysis [15–18, 20–23]. According to another study, there is a higher percentage of pruritus in patients with stage 3 (18%), stage 4 (26%) and stage 5 (42%) CKD, while the incidence patients with stage 5 CKD stage 5 on maintenance hemodialysis: was reported to be 58% [24]. Pruritus can be generalized, which is less frequently seen, and affects the back, face, and fistula arm [25]. However it can be localized to the back, abdomen, or at the head [26]. CKD-associated pruritus is linked to poor quality of life, sleep disturbance, and depression [27, 28]. Severe pruritus can also lead to depression [25], and may result in the development of skin excoriations due to excessive scratching [29]. There is also an increased risk of suicidal thoughts [30], and a higher risk of mortality [16, 25].

It has been found that the treatment of pruritus is affected by two factors: whether it is localized or generalized; and the severity. For measurement of the former, several tools can be used, such as the Visual Analogue Scale (VAS) [16], Eppendorf Itch Questionnaire (EIQ) [31], Skindex-10 [20], or itch MOS (itch medical outcome study) [20]. There is currently no universal scale adopted to measure CKD-associated pruritus’ prevalence, severity, and impact on quality of life. Indeed a universal scale should have the ability to evaluate the severity, degree, duration, and impact on quality of life in a heterogeneous patient population. In Pakistan the prevalence of chronic kidney diseases and CKD associated pruritus reported is 64.64–77.7% [32, 33] and the 5D itch scale is not validated in Pakistan. As English is not understood and spoken by majority of Pakistani population and Urdu language is the national language so it is widely spoken in Pakistan.

Therefore, the aim of our study was to validate the Urdu version of the 5D itch scale among patients with chronic renal disease in Pakistan.

## Method

A validation study was performed from July to September 2016, at a tertiary health care setting in Peshawar Pakistan. To assess for discriminative validity of the 5D–IS, we recruited patients regardless of whether or not they had pruritus.

### Participants

Patients aged 21 years and above, diagnosed with end stage renal disease (ESRD), and who were able to answer the questionnaire in Urdu were included. Participants with autoimmune diseases e.g. systemic lupus erythematous, and those having liver complications (e.g., hepatitis A, B or C) were excluded.

### Sample size

Based upon the ratio between the number of items in the scale to participants, which is 1:10 [34], the minimum sample size calculated was 50 participants.

### Instrument for translation

**Fig. 1:**
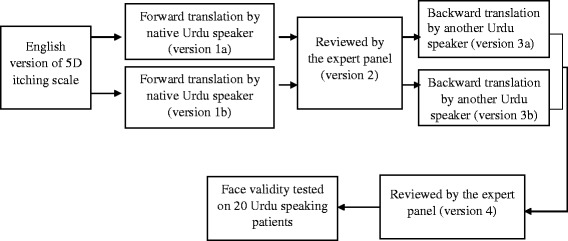
Flow chart of translation of 5D itch scale from English to Urdu

The 5D–IS originally developed by Elman et al. [31], is composed of five dimensions that address the duration, degree, direction, disability, and distribution of itching. For linguistic validation of the 5D–IS, the same five dimensions were translated into Urdu according to guidelines [35, 36] as shown in [Fig. 1, Additional file 1].

### Procedure

Participants were approached whilst they were undergoing dialysis. The objective of the study was explained, and written informed consent was obtained. To assess for reliability, the Urdu 5D–IS was administered to the same participants two weeks later.

### Statistical analysis

All analyses were performed using SPSS version 20.0 (SPSS Inc., Chicago, IL) at a significance level of *p* < 0.05. The normality of data was examined using skewness and kurtosis. Since the data was not normally distributed, non-parametric tests were used. Continuous variables were presented as median and interquartile range, however, categorical variables were presented as number and frequency. Exploratory factor analysis was performed to examine the construct validity of the 5D–IS. The Eigenvalues for a factor greater than one were considered as having a significant contribution in explaining the overall model variation, and were retained. Moreover, sampling adequacy was examined using Bartlett’s test of sphericity, and Kaiser–Mayer–Olkin (KMO). A KMO value greater than 0.6 suggests an acceptable level, while 0.80 indicates a good level of compatibility of the variables within the test [37]. For determining the discriminative validity, the Mann-Whitney U test was used to assess the score between ESRD on dialysis with pruritus or without pruritus at baseline, and re-test for no significant difference between test and retest results. For examining internal consistency Cronbach’s alpha was used, with a value more than 0.9 graded as Excellent, greater than 0.8 as Good, more than 0.70 as Acceptable, and greater than 0.6 graded as Questionable [38]. Furthermore, reliability was assessed using the intraclass correlation coefficient (ICC) and Kappa. An ICC value of ≥0.7.14 is acceptable [39], while Kappa values from 0.41–0.60 implies moderate agreement, 0.61–0.80 indicates substantial agreement, while 0.81–0.99 represents perfect agreement [40].

## Results

A total of 50 participants undergoing dialysis were recruited. Of these, 64% were males, while pruritus was reported by 70% of respondents (Table 1).

**Table 1:** Demographic characteristics of participants of Urdu 5D itch scale (*N* = 50)

Demographics	N (%)
Gender	
Male	32 (64)
Female	18 (36)
Median Age (years) and IQR	
Median	38.00
[IQR]	[30.75–52.75]
Education	
Illiterate	16 (32)
Primary level [6 years of education]	8 (16)
Secondary level [12 years of education]	10 (20)
College/Diploma [15 years of education]	7 (14)
Tertiary [≥16 years of education]	9 (18)
Marital status	
Single	18 (36)
Married	32 (64)
Dependent on family	34 (68)
Expenses for treatment borne by	
Government	33 (66)
Family	17 (34)
Monthly income	
Dependent on family	19 (38)
Less than PKR 30,000	14 (28)
PKR 30,000 to 51,000	14 (28)
PKR 51,000 to 75,000	2 (4)
PKR 75,000 and above	1 (2)
Duration of having kidney disease (years)	3 months −10 years
Pruritus recorded in patients	35 (70)

### Construct validity (factor analysis)

Exploratory factor analysis revealed that the 5D–IS had 2-factor loadings: “Pattern and activity” and “Distribution” (Table 2), which explained 76.3% of the variance [KMO = 0.802, Chi-Square = 297.704, Bartlett’s test of sphericity was significant (*df* = 28, *p* < 0.001)].

**Table 2:** Item Factor Loading based of Principal Component Analysis with Varimax Rotation of Urdu 5D itch scale

Domains	Factor 1 Pattern and activity	Factor 2 Distribution
Duration	0.754	
Degree	0.778	
Direction	0.674	
Sleep	0.646	
Social	0.892	
House work	0.914	
School/work	0.838	
Distribution		0.923

### Discriminative validity

Discriminative validity was assessed using the Mann-Whitney U test to assess if there was no significant difference between baseline and retest results. The median scores for the 5D itch scale for all five domains at baseline (*n* = 50), and re-test (*n* = 47) are shown in Table 3. This indicates that participants interpreted the items similarly at baseline and retest, and the scale is therefore stable, as shown in Table 3.

**Table 3:** Score of patients at test (Baseline) and re-test Urdu 5D itch scale

Domains	Test (*n* = 50)		Re-test (*n* = 47*)*		Mann-Whitney U test
	Median	IQR	Median	IQR	*p* value
Duration	1.00	0.00–2.00	1.00	0.00–2.00	<0.001*
Degree	3.00	0.00–3.00	3.00	0.00–3.00	<0.001*
Direction	2.00	0.00–3.00	2.00	0.00–3.00	<0.001*
Disability					
Sleep	1.00	0.00–3.00	1.00	0.00–3.00	<0.001*
Leisure/Social life	1.50	0.00–3.00	2.00	0.00–3.00	<0.001*
House work	1.00	0.00–3.00	1.00	0.00–3.00	<0.001*
Work/school	1.00	0.00–2.00	1.00	0.00–2.00	<0.001*
Distribution	1.00	1.00–2.00	1.00	1.00–2.00	0.002 *

*IQR* interquartile range; * statistically significant at *p* < 0.05

### Reliability

The overall Cronbach’s alpha for the 5D–IS Urdu version was 0.914, with significant intraclass correlation coefficient (*p* < 0.001). At retest, three participants were lost to follow up as they did not want to answer the questionnaire again. The intraclass correlation coefficient (ICC) between the 5D itch scale test re-test was 0.9160 (95% confidence interval 0.941–0.975), a highly significant correlation (*p* < 0.0001). The overall Kappa value was 0.900, which was significant (*p* < 0.001), while the individual kappa value is shown in Table 4.

**Table 4:** The psychometric properties of the Urdu 5D–Itch Scale

Domains	Test Median	Re-test Median	Corrected Item total Correlation	Cronbach’s Alpha if Item Deleted	Kappa Value
Duration	1.00	1.00	0.764	0.900	1.000
Degree	3.00	3.00	0.882	0.888	0.940
Direction	2.00	2.00	0.796	0.898	0.944
Disability					
Sleep	1.00	1.00	0.668	0.909	1.000
Leisure/Social life	1.50	2.00	0.763	0.900	1.000
House work	1.00	1.00	0.751	0.901	1.000
Work/school	1.00	1.00	0.784	0.899	1.000
Distribution	1.00	1.00	0.355	–	1.000

Thighs, Buttock and Abdomen were the most affected body parts reported by respondents

## Discussion

Pruritus is one of the most common symptoms observed in patients with chronic kidney disease undergoing dialysis. Previous studies have presented different prevalence rates of CKD-associated pruritus, ranging from 22% to 84% among patients undergoing dialysis [41–43]. Our study found a prevalence rate of 70% in Pakistan, which is almost similar to previous studies in Pakistan which reported prevalence rates of 64% [44], 64.64% [33], and 77.7% [32]. The intensity of pruritus as reported in our study was mild (14%), moderate (50%), and severe (14%); differing from a previous study in Pakistan which reported different pattern of intensity mild (50%), moderate (28%), and severe (14%) [45]. The current study established that CKD-associated pruritus has a distressing impact, and contributes to lower quality of life. Important aspects related to quality of life affected by CKD-associated pruritus in this study were delay in falling asleep, and impaired ability to perform housework/errands. Similar finding were also found in other studies in which patients with moderate to extreme intensity of CKD-associated pruritus reported difficulty sleeping at night, feeling sleepy during the day, and nocturnal awakenings [46, 47]. The onset, duration, and the intensity of pruritus can change over time, and the itching is usually worse at night [25]. Pruritus may be episodic or constant, may be generalized or localized, and its intensity may vary from mild to severe [48]. Among patients with localized pruritus, the body parts most affected are the back, limbs, chest, and head; while about 20–50% of patient experience generalized pruritus [25]. However, our findings suggest that among the sixteen body parts listed in the Urdu version of the 5D itch scale, the following body parts i.e. thighs, buttocks, and abdomen, were the most effected; as reported by respondents.

The Visual analogue scale (VAS) is the most frequently used tool for quantification of pruritus. VAS is an adequate tool for assessing the severity of pruritus, however, it failed to address other aspects of pruritus like its relative impact on quality of life. Other tools recommended by the International Forum for the Study of Itch (IFSI) [49] for assessment of CKD-associated pruritus in patients undergoing dialysis are the itch medical outcome study [20], Skindex-10 [20] and brief itchy inventory [20]. However, these tools lack a comprehensive assessment of its psychometric properties. In order to address the short comings of these tools, a multidimensional tool i.e. the 5D itch tool, a single page tool which was specifically designed for measuring itch/pruritus that is easy to complete, assesses the impact of itch on quality of life, and adequately detects changes over time [31].Indeed the 5D itch scale has the ability to evaluate the severity, degree, duration and impact on quality of life in domains such as sleep, leisure/social life, and house work. The internal consistency of the English version of the 5D itch scale was good i.e. Cronbach’s alpha = 0.734 [31]. However, the Cronbach’s alpha for the Urdu version was found to be higher (Cronbach’s alpha = 0.914 with significant intraclass correlation coefficient *p* < 0.001). In our study, the KMO value was 0.802 with Chi-Square value (χ2 = 297.704), and Bartlett’s test of sphericity was significant (*df* = 28, *p* < 0.001). The intraclass correlation coefficient (ICC) between the 5D itch scale test re-test was 0.939 (95% confidence interval 0.91–0.96), which is a highly significant correlation (*p* < 0.0001), while for the English version of the 5D itch scale the ICC obtained was 0.96 (95% confidence interval 0.92–0.98), which is also a highly significant correlation *p* < 0.0001 [31]. The overall Kappa value calculated is 0.742, which was also significant (*p* < 0.001). Also, the discriminative validity established no significant changes in test (baseline) and re-test results.

### Strengths and limitations

The Urdu version of the 5D itch scale may be a useful tool in clinical trials of patients with chronic kidney disease in Pakistan where pruritus is assessed. It can also be used in further large-scale studies involving different etiologies of pruritus, to assess itch-severity and patient response to treatment by clinicians, dermatologists, and nephrologists. The only limitation of this study is that only patients with ESRD (Stage 4–5 CKD patients) were recruited.

## Conclusion

The results showed that the Urdu version of the 5D itch scale had good reliability and validity for evaluating pruritus among patients with chronic kidney disease in Pakistan. Therefore, for clinical studies and assessment of pruritus t in patients with chronic kidney disease, the Urdu version of the 5D itch scale is a suitable and brief tool that can be used.

## Additional file

Additional file 1: Urdu 5D itch scale. (DOCX 19 kb)

## Abbreviations

5D–IS: 5D itching scale
CKD: Chronic kidney disease
EIQ: Eppendorf itch questionnaire
ESRD: End stage renal disease
HD: Hemodialysis
ICC: intraclass correlation coefficient
KMO: Kaiser–Mayer–Olkin
MUHREC: Monash University Human Research and Ethics Committee
PD: Peritoneal dialysis
VAS: Visual analogue scale
