# Supplementary material for: Validity and reliability of the Urdu version of the 5D itching scale to assess pruritus among patients with chronic kidney disease in Pakistan
Source: BMC Nephrol. 2017 Oct 2;18:302. doi: 10.1186/s12882-017-0717-0 (PMC5625599; doi:10.1186/s12882-017-0717-0)
Supplement: Additional file 1: — Urdu 5D itch scale. (DOCX 19 kb) [file 12882_2017_717_MOESM1_ESM.docx]

-**5 اردو ڈی ایچنگ سکیل**

**۱۔دورانیہ:پچھلے دو ہفتوں کے دوران روزانہ کے حساب سے آپ کو اوسطاً کتنے گھنٹے خارش کی تکلیف رہی ہے۔؟**

**چھ گھنٹے سے کم۔۔۔۔۔۔۔۔۔۔۔۔ ۶گھنٹہ تا ۱۲ گھنٹے ۔۔۔۔۔۔۔۔۔۔۔۔۔ ۱۲تا ۱۸گھنٹے۔۔۔۔۔۔۔۔۔۔۔۔۔**

**۱۸تا۲۳گھنٹے۔۔۔۔۔۔۔۔۔۔۔۔ تما م دن۔۔۔۔۔۔۔۔۔۔۔۔۔۔۔۔۔۔**

**۲۔کیفیت/درجہ:براہِ مہربانی گذشتہ دوہفتے کے حساب سے خارش کی تکلیف کی شدت کا تعین کیجئے ۔**

| **کوئی تکلیف نہیں تھی** | **معمولی اورہلکی سی تھی** | **درمیانے درجہ کی تھی** | **شدید قسم کی تھی** | **ناقابلِ برداشت حد تک تھی** |
| --- | --- | --- | --- | --- |
| **1** | **2** | **3** | **4** | **5** |

**۳۔گذشتہ دوہفتوں کے مقابلے میں خارش کی تکلیف مکمل طور پرختم ہوگئی یااس میں بہتری آگئی یا پھر اس میں بدترین حد تک اضافہ ہوا۔**

| **مسئلہ مکمل طور پرحل ہوگیا** | **حالت بہت بہتر ہوئی ہے لیکن مسئلہ تاحال موجودہے** | **حالت تھوڑی بہترہوئی لیکن مسئلہ تاحال موجودہے** | **کوئی فرق نہیں پڑا** | **حالت بدترہوتی جارہی ہے** |
| --- | --- | --- | --- | --- |
| **1** | **2** | **3** | **4** | **5** |

**۴۔پچھلے دوہفتوں کے تجربے کی بنیاد پر درج ذیل سرگرمیوں پر خارش کے اثرات کا اندازہ لگائیے ۔**

**نیند:**

| **خارش کا مسئلہ کبھی بھی نیند پر اثرانداز نہیں ہوا** | **کبھی کبھار نیند دیر سے آتی ہے** | **نیند روزانہ کافی دیر سے آتی ہے** | **نیند بھی کافی دیر سے آتی ہے اور کبھی کبھار رات کو تکلیف کی وجہ سے نیند میں خلل واقع ہوتا ہے** | **نیند بھی کافی دیر سے آتی ہے اور تسلسل کے ساتھ تکلیف کی وجہ سے نیند میں خلل واقع ہوتاہے** |
| --- | --- | --- | --- | --- |
| **1** | **2** | **3** | **4** | **5** |

**تفریح /سماجی :**

| **N/A** | **اس تکلیف سے میرے سماجی مشاغل کبھی بھی متاثر نہیں ہوتے** | **اس تکلیف سے میرے سماجی مشاغل کبھی کبھارمتاثر ہوتے ہیں** | **اس تکلیف سے خاص مواقع پر میرے سماجی مشاغل متاثر ہوتے ہیں** | **اس تکلیف سےہمیشہ میرے سماجی مشاغل متاثر ہوتے ہیں** |
| --- | --- | --- | --- | --- |
| **1** | **2** | **3** | **4** | **5** |

**گھر یلوکام/کسی مقصد کے لیے گھر سے باہر نکلنا:**

| **N/A** | **کبھی بھی متاثر نہیں ہوتے** | **کبھی کبھارمتاثر ہوتے ہیں** | **خاص مواقع پر متاثر ہوتے ہیں** | **ہمیشہ متاثر ہوتے ہیں** |
| --- | --- | --- | --- | --- |
| **1** | **2** | **3** | **4** | **5** |

**کام /سکول :**

| **N/A** | **کبھی بھی متاثر نہیں ہوتے** | **کبھی کبھارمتاثر ہوتے ہیں** | **خاص مواقع پر متاثر ہوتے ہیں** | **ہمیشہ متاثر ہوتے ہیں** |
| --- | --- | --- | --- | --- |
| **1** | **2** | **3** | **4** | **5** |

**۵۔تقسیم :**

**گذشتہ دو ہفتوں سے آپ کے جن اعضاء میں خارش کی شکایت رہی ہے ان کی نشاندہی کریں ۔اگر کسی متعلقہ عضو کا ذکر نہیں کیاگیاہے تو اس عضو کی نشاندہی کریں جو علمِ تشریح الابدان کی رو سے قریب تر ہو۔**

| **عضو کا نام** | **√** |  | **√** |  | **√** |  | **√** |
| --- | --- | --- | --- | --- | --- | --- | --- |
| **سر/کھوپڑی کی جلد** |  | **کمر** |  | **تلوے** |  | **کہنیوں سے اوپر بازو کا حصہ** |  |
| **چہرہ** |  | **سرین** |  | **ہتھیلیاں** |  | **زیریں لباس یا کمر پٹی کے ساتھ لگنے والے جسم کے حصے** |  |
| **سینہ** |  | **ران** |  | **ہاتھوں کا اوپر والاحصہ /انگلیاں** |  | **ران کے اوپر کا جوڑ** |  |
| **پیٹ** |  | **پاؤں کے نیچے والے حصے** |  | **کہنی سے کلائی تک کا بازو** |  | **پیر کے اوپر والے حصے /انگلی** |  |
